# Work-to-rest ratios in repeated sprint assessments: A replication study

**DOI:** 10.1055/a-2940-7047

**Published:** 2026-09-04

**Authors:** Lauren B. L. Jutlah, Kailey A. Stevens, Ashley Meelu, Jeremy N. Cohen, Jennifer Murphy, Joe P. Warne, Jason S. Au

**Affiliations:** 1 Department of Kinesiology and Health Sciences 8430 University of WaterlooWaterlooONCanada; 2 School of Biological, Health and Sport Sciences 8819 Technological University DublinDublinLeinsterIreland

**Keywords:** sprint testing, cycling performance, fatigue, reproducibility

## Abstract

Replication projects provide supplementary evidence to reinforce the veracity of novel claims. The Sports Science Replication Centre conducted an international effort to assess the replicability of sports and exercise science research; our research group was assigned to replicate the study by La Monica et al (2016) who reported that larger work-to-rest ratios during repeated cycle sprinting elicited greater oxygen consumption during the rest periods. Participants ( *n* = 21 men, 23 ± 3 y) completed a familiarization and three experimental visits of 10 maximal 6-second cycle sprints against a resistance of 7.5% body weight separated by one of the three active rest intervals: 24 seconds (1:4 work-to-rest ratio), 18 seconds (1:3), or 12 seconds (1:2). Both V̇O _2work_ and V̇O _2rest_ were higher during the 12-second rest interval compared to the 18-second and 24-second rest intervals ( *p* < 0.01). Changes in V̇O _2rest_ were similar to the original study, although our observed effect size was lower (partial *η* ^2^ = 0.41 compared to the original partial *η* ^2^ = 0.75), consistent with typical replication studies. We observed differences in V̇O _2work_ unlike the original study which found no differences between work-to-rest conditions. This replication attempt was partially successful in corroborating previous findings, supporting that larger work-to-rest ratios lead to decreased sprint performance and larger V̇O _2_ demands, with more conservative effect sizes.

## Introduction

The replication crisis describes the phenomenon in modern science in which repeating the findings of certain research fields is becoming increasingly difficult to achieve. 1 2 3 This trend was initially described in psychology and initial analyses suggest that study design, statistical analyses, statistical interpretations, and publishing bias contribute to a high degree of type I (false-positive) error in published findings. 3 4 5 Although the replication crisis has taken prominence in the behavioral sciences, there is reason to believe that sports and exercise science are susceptible to parallel issues due to similar experimental designs, inferential statistics, and small sample sizes in human studies. 6 7 In response to the ambiguity in the replicability of sports science research, 8 9 the Sports Science Replication Centre has established an international effort to systematically evaluate the replicability of sports science 10 11 12 by allocating replication projects to independent sports science laboratories. Our research group is a participant in this effort and was randomly allocated a viable published study that aligned with our available methodological resources (for a detailed description of the randomization process, please refer ref. 13 ). In addition to the larger replication project, individually reporting the findings of each replication study can also add value to the specific area of the study in question, and therefore, the purpose of this paper is to report the specific replication findings of La Monica et al, 14 for their paper titled ‘Altering work-to-rest ratios differentially influences fatigue indices during repeated sprint ability (RSA) testing.’

La Monica et al 14 conducted a within-subject, repeated measures study to examine the influence of work-to-rest ratios on oxygen consumption, performance (total work [TW], peak power [PP], and mean power [MP]), and fatigue indices. The motivation for the study was to support the development of sprint ability tests that can be tailored to an athlete’s competitive environment. The original study investigated eight male participants who performed three RSA protocols on a cycle ergometer. The protocol consisted of 10 maximal 6-second cycle sprints against a resistance of 7.5% of their body weight (BW) separated by one of the three active rest intervals: 24 seconds (1:4 work:rest ratio), 18 seconds (1:3), or 12 seconds (1:2). Using methods available in their report, we sought to replicate the reported results and effect sizes for oxygen consumption (V̇O _2_ ) and fatigue indices from the original paper.

## Materials and methods

### Ethics approval

The study protocol received ethics clearance through the University of Waterloo Research Ethics Committee (ORE #44277). The research methods and protocols adhere to the recommendations outlined by the Declaration of Helsinki concerning the use of human participants. All participants provided written informed consent prior to engaging in any study protocols.

### Participants

We recruited 21 healthy men between the ages of 19 and 30 (23 ± 3 y; 178 ± 5 cm; 78.6 ± 7.9 kg) and who were recreationally active, engaging in moderate-to-vigorous physical activity at least twice per week. Inclusion criteria were identical to the original report. Multiple methods were used for power analysis and to determine the sample size as per the Sports Science Replication Centre’s protocol. 13 In addition, only one dependent variable was selected for the larger replication project, which was V̇O _2rest_ for this replication study. Therefore, we used a partial eta square of 0.751 reported for V̇O _2rest_ in the original study. Due to the overinflation of original effect sizes with smaller sample sizes, 7 we first used the bias and uncertainty corrected sample size method 15 which estimated a sample size of 12 participants to be recruited for a power of 95% at an assurance level of 95%. However, we then determined the minimum target sample size was *n* = 16 by doubling the original sample size ( *n* = 8) as this was feasible to recruit. See the project page on the Open Science Framework for full details of sample size calculations (https://doi.org/10.17605/OSF.IO/2DJZM). An additional five participants were recruited to protect data-dropout as the list-wise exclusion was used during data analysis. Exclusion criteria included daily cigarette smokers and current or past diagnosis of cardiovascular, respiratory, metabolic, neurological, or orthopedic disorders that could interfere with cycle ergometer performance. All inclusion and exclusion criteria matched that provided by La Monica et al. 14

### Study design and protocol

The study methods were replicated from La Monica et al 14 and were pre-registered on the Open Science Framework prior to data collection ( https://osf.io/y6ngr ). The study had a within-subject repeated measure design to compare the physiological (V̇O _2_ ) and performance (TW, PP, and MP) responses during RSA testing on a cycle ergometer. Participants completed four experimental visits; familiarization followed by three experimental visits of 10 maximal 6-second cycle sprints against a resistance of 7.5% of their BW separated by one of three unloaded active rest intervals: 24 seconds (1:4 work:rest ratio), 18 seconds (1:3), or 12 seconds (1:2). Familiarization was conducted identical to the original study, where participants practiced 10 maximal 6-second sprints followed by 24- second rest under testing conditions. Each experimental visit started with a 4-minute warm-up at a self-selected pace with four sprints interspersed lasting 4 to 6 seconds each. This was followed by three 6-second maximal sprints against a load of 7.5% BW with 24 seconds of unloaded cycling between to establish PP. Participants then remained on the cycle ergometer for 5 minutes of passive rest before the 10 maximal sprints. Prior to each sprint during the active rest phase, the researcher counted down the participant where they would cease cycling to position their pedals into the starting position with their dominant foot forward at a 45° angle to the vertical axis. For each sprint, participants received strong verbal encouragement to perform maximally. Participants were asked to maintain their regular dietary and nutritional intakes prior to each testing day, confirmed by a diet log. Study visits were separated by at least 48 hours, and participants were asked to refrain from any vigorous exercise 48 hours prior to the testing day to avoid influence from soreness and fatigue.

### Experimental measures

#### Oxygen uptake

Gas exchange analysis was performed throughout the test using a breath-by-breath metabolic cart (Sensormedics Vmax Spectra 22 Pulmonary Function System, Yorba Linda, CA, USA), measured as V̇O _2work_ and V̇O _2rest_ representing the maximum V̇O _2_ during the sprint and the minimum V̇O _2_ during the active rest, respectively. The flowmeter was calibrated 20 minutes prior to testing for each subject. Gas analyzers were calibrated daily with room air and gases of known concentration. A one-way valve was stabilized using a mouthpiece and a head unit to measure expired oxygen and carbon dioxide flow rates.

#### Performance

Performance was evaluated by PP (the highest power output achieved during a 6-s sprint), TW (the accumulated work during a 6-s sprint, calculated from the MP and length of the sprint), and MP (the average power output maintained throughout a 6-s sprint). Performance data were collected using PowerLab (AD Instruments Inc, Colorado Springs, CO, USA) to display in LabChart software (LabChart v7.3.8, AD Instruments). Fatigue indices were determined as the percent decrement (%Dec) and the rate of decline. The percent decrement was calculated using the formula in eqn 1 for PP, MP and TW.



Fatigue as measured by the rate of decline was calculated as the slope coefficient of the line of best fit that was generated by the regression line between the number of sprints completed and PP, MP and TW.

### Statistical analysis

All analyses and statistics followed the original procedures outlined by La Monica et al. 14 Breath-by-breath gas exchange data were offloaded as Excel files and analyzed using RStudio (v4.3.0). Data were fitted using a cubic spline interpolation function and plotted over time for each trial. Maximum and minimum values were determined for each RSA test as V̇O _2work_ and V̇O _2rest_ , respectively. A low pass Butterworth digital filter with a cut-off frequency of 0.06 Hz was used to smooth the V̇O _2_ data. V̇O _2work_ and V̇O _2rest_ for each RSA trial were averaged to compare the physiological responses between the work to rest ratios.

Differences between trials were determined using SPSS (version 29; SPSS Inc., Chicago, IL, USA), with *α* = 0.05. Normality was confirmed via the Kolmogorov–Smirnov test and parametric statistics were conducted. A repeated measures analysis of variance (ANOVA) was used to analyze differences in 24-second, 18-second and 12-second work:rest intervals, with least significant difference post-hoc analysis for statistical significance. Partial *η* ^2^ is reported for the omnibus ANOVA effect. We compared the original and replication effect size estimates using *z* -tests to determine if the replication and original effect size estimates were statistically different using the “TOSTER” R package (version 0.5.0). 16 Interpretations of the significance tests and *z* -tests were completed according to the guidelines of Brandt et al. 17 We also compared the direction and significance of the Omnibus ANOVA effect between the original and replication results.

## Results

### Oxygen uptake

**Fig. 1:**
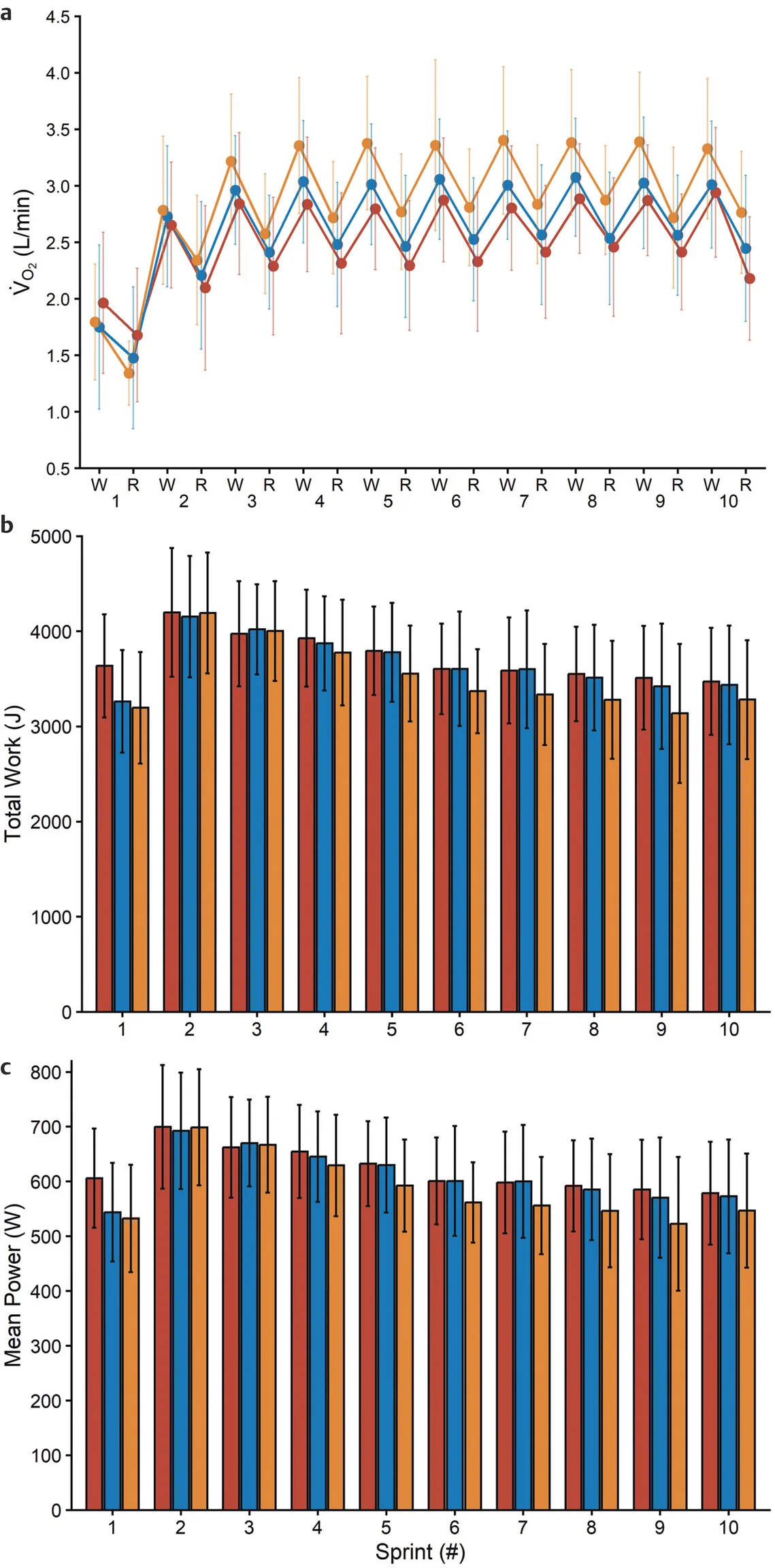
Changes in ( **a** ) oxygen consumption (W = work and R = rest), ( **b** ) total work, and ( **c** ) mean power over the course of the 10 repeated sprints for the 24-second trial (red), 18-second trial (blue), and 12-second trial (yellow) conditions. Mean ± SD is shown in line and bar graphs.

V̇O _2_ values for the 10 sprints during the RSAs are shown in Figure 1 and presented in Table 1 . V̇O _2work_ was altered with different work-to-rest intervals ( *F* (1,20) = 21.97, *p* < 0.001, and *η* ^2^_p_ = 0.52), where V̇O _2work_ was greater in the 12-second trial compared to the 24-second ( *p* < 0.001) and 18-second trials ( *p* < 0.001) and was greater in the 18-second trial compared to the 24-second trial ( *p* = 0.017). V̇O _2rest_ was also impacted by the work-to-rest intervals ( *F* (1,20) = 13.82, *p* < 0.001, and *η* ^2^_p_ = 0.41), where V̇O _2rest_ was greater in the 12-second trial compared to the 24-second ( *p* < 0.001) and 18-second trials ( *p* < 0.001) without difference between the 18-second and 24-second trials ( *p* = 0.060).

**Table:** **Table 1** Comparison of physiological and performance variables ( *n* = 21)

	12 s	18 s	24 s
V̇O _2work_ (L·min ^−1^ )	3.14 ± 0.53*†	2.87 ± 0.48*	2.75 ± 0.48
V̇O _2rest_ (L·min ^−1^ )	2.57 ± 0.45*†	2.37 ± 0.49	2.25 ± 0.52
Average TW (J)	3513 ± 448*†	3667 ± 500	3726 ± 421
Average MP (W)	585 ± 75 *†	611 ± 83	621 ± 70
Average PP (W)	711 ± 89*†	744 ± 106*	766 ± 98
Percent decline			
TW (%)	−18.11 ± 6.83*†	−13.90 ± 6.53	−13.40 ± 6.83
MP (%)	−18.11 ± 6.83*†	−13.90 ± 6.53	−13.40 ± 6.83
PP (%)	−16.34 ± 7.55*†	−12.06 ± 6.05	−13.03 ± 5.80
Rate of decline			
TW (J·sprint ^−1^ )	−71.18 ± 81.01	−43.05 ± 63.99	−58.22 ± 77.74
MP (W·sprint ^−1^ )	−11.86 ± 13.50	−7.17 ± 10.67	−9.70 ± 12.96
PP (W·sprint ^−1^ )	−22.77 ± 15.47*†	−17.56 ± 12.27	−19.00 ± 14.20

Abbreviations: MP, mean power; PP, peak power; TW, total work.

*Significantly different ( *p* ≤ 0.05) from the 24-second trials; ^†^ Significantly different ( *p* ≤ 0.05) from the 18-second trials.

### Cycling performance

Participants were similarly sized compared to the original study report (78.6 ± 7.9 vs. 75.1 ± 10.9 kg). The average MP and TW for the 10 sprints during the RSAs are shown in Figure 1 . All performance values are outlined in Table 1 . The TW varied with work-to-rest intervals ( *F* (1,20) = 7.46, *p* < 0.001, *η* ^2^_p_ = 0.27), where the TW was greater in the 18-second ( *p* = 0.008) and 24-second ( *p* = 0.002) trials compared to the 12-second trial. The MP was similarly affected by work-to-rest intervals ( *F* (1,20) = 7.46, *p* < 0.001, *η* ^2^_p_ = 0.27), where the MP was greater in the 18-second ( *p* = 0.008) and 24-second ( *p* = 0.002) trials compared to the 12-second trial. Differences in PP values were also observed between each work-to-rest intervals ( *F* (1,20) = 14.85, *p* < 0.001, *η* ^2^_p_ = 0.43), where the PP during the 18-second trial was greater than the 12-second ( *p* = 0.002), and the PP in the 24-second trial was greater than the 18-second ( *p* = 0.035) and 12-second trials ( *p* < 0.001).

### Fatigue indices

Fatigue index values are presented in Table 1 . PP %Dec during RSA varied with shorter work-to-rest intervals ( *F* (1,20) = 9.50, *p* < 0.001, *η* ^2^_p_ = 0.32), where PP %Dec was greater in the 12-second trial compared to the 18-second ( *p* < 0.001) and 24-second trials ( *p* = 0.004). Similar patterns were found with MP %Dec ( *F* (1,20) = 11.73, *p* < 0.001, *η* ^2^_p_ = 0.37), where MP %Dec was greater in the 12-second trial compared to the 18-second ( *p* < 0.001) and 24-second trials ( *p* < 0.001). TW %Dec results also changed with shorter work-to-rest intervals ( *F* (1,20) = 11.73, *p* < 0.001, *η* ^2^_p_ = 0.37), where TW %Dec was greater in the 12-second trial compared to the 18-second ( *p* < 0.001) and 24-second trials ( *p* < 0.001).

The rate of decline was affected by changes in work-to-rest intervals. The PP rate of decline values increased with shorter work-to-rest intervals ( *F* (1,20) = 4.06, *p* = 0.03, *η* ^2^_p_ = 0.17), where the PP rate of decline of the 12-second trial was larger than the 18-second ( *p* = 0.03) and 24-second trials ( *p* = 0.04). Neither the MP ( *F* (1,20) = 2.70, *p* = 0.08, *η* ^2^_p_ = 0.12) nor the TW ( *F* (1,20) = 2.70, *p* = 0.08, *η* ^2^_p_ = 0.12) rate of decline values changed with shorter work-to-rest intervals.

### Replication of original effects

The original study reported no differences in V̇O _2work_ across conditions and a stepwise increase in V̇O _2rest_ with decreasing work-to-rest ratios. In contrast, we observed elevated V̇O _2work_ and V̇O _2rest_ only in the 12-second rest conditions with no differences between the 18-second and 24-second rest conditions. With respect to power, the original study did not observe differences in the PP between conditions and only observed differences in the MP and TW between the 12-second and 24-second conditions. In contrast, we observed stepwise decreases in the PP with decreasing work-to-rest intervals, and discrimination of MP and TW between 12-second and both 18-second and 24-second conditions. Our %Dec and rate of decline results were likewise more sensitive to work-to-rest ratios, with clear differences between the 12-second and longer rest interval conditions, whereas the original study only identified differences in the rate of decline outcomes.

Our effect sizes for omnibus ANOVAs ranged from 0.12 to 0.52 (median = 0.32), which were slightly tighter than the original study range of 0.02 to 0.75 (median = 0.34; 14 Table 2 ). The replication effect size estimate ( *η*_p_^2^ = 0.41) was significantly smaller than the original for V̇O _2rest_ ( *η*_p_^2^ = 0.75; *z* = 1.96, *p* = 0.03). Therefore, we judge it a “practical failure to replicate” as per the replication recipe 17 because it was significantly different from the null and the replication effect size estimate was significantly different from the original, and therefore not compatible. The replication effects for V̇O _2work_ ( *z* = −2.65, *p* = 0.99), PP ( *z* = −3.77, *p* = 0.99), MP ( *z* = 0.90, *p* = 0.18), and TW ( *z* = 0.77, *p* = 0.22) were not significantly different from the originals and were therefore comparable on replication.

**Table:** **Table 2** Comparison of reported effect sizes (partial *η* ^2^ ) of the original and replication effort

Outcome	Original effect size	Replication effect size
V̇O _2work_	0.10	0.52
V̇O _2rest_	0.75	0.41
Average TW	0.41	0.27
Average MP	0.44	0.27
Average PP	0.02	0.43
**Percent decline**		
TW	0.24	0.37
MP	0.10	0.37
PP	0.32	0.32
**Rate of decline**		
TW	0.46	0.12
MP	0.40	0.12
PP	0.36	0.17

Abbreviations: MP, mean power; PP, peak power; TW, total work.

## Discussion

We aimed to replicate the findings of La Monica et al, 14 where V̇O _2rest_ and fatigue indices increase with shorter work-to-rest ratios during repeated sprint cycling. We observed a clear effect for short (12 s) rest periods to increase V̇O _2_ and fatigue indices, and a stepwise response for decreasing rest periods to impair performance over the course of a repeated cycle sprint protocol. Both studies generally conclude that larger work-to-rest ratios (i.e., 1:2 > 1:3 > 1:4) lead to decreased performance and larger V̇O _2_ demands, with comparable effect sizes throughout. 14 While the overarching message is the same, we observed slight differences in the strength of observations and whether longer rest periods longer than 12 seconds have a measurable impact on performance in recreational male cyclists, highlighting the benefit of replication studies to evaluate previous results in sports sciences.

### Changes in oxygen uptake in response to decreased work-to-rest ratios

We observed a clear effect of short rest periods (12-s rest, 1:2 work:rest ratio) to increase V̇O _2work_ and V̇O _2rest_ during repeated cycle sprinting, although with a practical failure to replicate due to the smaller observed effect sizes. These results partially agree with the original results, which observed a stepwise increase in V̇O _2rest_ with shorter rest periods, but no effect on V̇O _2work_ . Mechanistically, V̇O _2_ is expected to remain higher after shorter rest periods due to a higher post-exercise oxygen consumption response. We were likely unable to detect a difference between the 18-second and 24-second recovery conditions due to non-linear V̇O _2_ recovery kinetics, resulting in a floor to the V̇O _2_ response with successive sprints. An advantage of this replication effort is increasing the sample size of individuals with similar inclusion/exclusion criteria to expand the true range of responses to a physiological task. Differences in our conclusions likely stem from power issues with the original study, whereas replication efforts can capture more realistic variability in responses for the same experimental protocol. This is also observed in a more consistent effect size between V̇O _2work_ and V̇O _2rest_ , whereas there was large variability in the original paper.

Generally, our V̇O _2work_ values were consistently lower than the original values and our V̇O _2rest_ values were consistently larger, whereas our performance (PP, MP, and TW) values were consistently lower (i.e., Δ -381 W, -150 W, and -793 J, respectively, in the 12-s trial). On average, our participants were similar to the original study’s participants (Δ +3.4 cm tall, +3.5 kg body mass), which we expected to translate to similar V̇O _2_ and performance values. While the inclusion criteria specified recreationally active men (≥2 d/wk of exercise), the type of training was not specified (i.e., aerobic vs. strength and cycling vs. running) which may have influenced their responses to cycling sprints.

### Changes in power and work in response to decreased work-to-rest ratios

Our MP and TW results were similar the original study with additional discrimination between the 12-second and 18-second trials. Unlike the original study which found no effect, we observed progressively decreasing PP with shorter rest periods. Reductions in power and work are expected with repeated sprints and reflect enhanced muscular fatigue with shorter rest periods. During repeated sprint cycling, there is a decrease in EMG amplitude that parallels the decrease in power output, despite maximal effort. 18 19 Up to 30-second rest is necessary to regenerate the same power output for three to four consecutive 6-second sprints. 18 20 21 Of note to the replication, the TW and MP values of the first sprint in this study were always less than the values for the second sprint, after which the expected decline occurs. Our replication effort incorporated methodological replication, where we relied on the study methods as written to replicate the findings. One concern that was raised was whether there was a ‘dead wheel’ (stagnant flywheel) to begin the first sprint of the protocol after the initial 5-minute passive rest phase. Without clarification, we chose to use a dead wheel, which likely resulted in the first sprint being of a lower TW due to the force required to overcome the initial inertia, versus an active wheel prior to cycle sprinting. Any deficit was recovered by the second sprint when the wheel was engaged. Differences between participants may be due to variances in the initial power produced by each participant, 18 possibly explaining the lower effect size noted for performance outcomes in the replication effort. Given the brief sprint efforts and that this protocol was used for all trials, it is unlikely that this choice impacted the comparison between the 12-second, 18-second and 24-second conditions as only averages of the conditions are reported, not the dynamic changes across the condition, as per the original methods.

### Limitations of the replication

Limitations in the general methodology of the original study may have introduced bias to the results of both investigations. The original study did not note randomization of the order of work-to-rest ratios; as such, each participant completed the study with progressively shorter rest periods which may have caused either learning effects of the sprint effort, or enhanced fatigue for the final 12-second rest conditions. We would have included a randomization process of the methods to avoid this bias. The study also excluded women which raises the question of whether the results are generalizable to both sexes. While we sourced a breath-by-breath metabolic cart to conduct the study, the model and manufacture of the assessment equipment differed between studies. There is the possibility that device differences account for some of the heterogeneity between study results, although we contend that between-subject variability based on the sample sizes are a more striking reason for the differences in effect sizes. Furthermore, the original study analyzed the average V̇O _2_ and power data across a full sprint protocol (averaged across 10 sprints), whereas an analysis of the data for individual sprint efforts may have been more powerful in observing dynamic responses to shorter rest intervals.

## Conclusion

We aimed to replicate the findings of La Monica et al 14 to contribute to the growing field-wide assessment of a replicability crisis in sports science. Although our methods were exact to those of the written methods in the original study, some aspects of the protocol made replication difficult. While we were able to generally replicate the overarching message of the paper (i.e., larger work-to-rest ratios lead to increased fatigue and larger V̇O _2_ demands), the size of the effect varied across outcomes and was significantly lower for V̇O _2rest_ , and we were able to detect additional sensitivity of very short (1:2 work:rest ratios) to fatigue metrics. Our findings contribute to the ongoing international effort to determine whether there is a replication crisis in the sports sciences, highlighting the need for detailed protocols and larger, more generalizable participant cohorts in exercise studies.

## Data availability statement

Raw data is available online at https://osf.io/db3sk/ .

## Declaration of generative AI use

The authors confirm that no Generative AI was used in the preparation of this manuscript.
